# A Monomeric Variant of Insulin Degrading Enzyme (IDE) Loses Its Regulatory Properties

**DOI:** 10.1371/journal.pone.0009719

**Published:** 2010-03-16

**Authors:** Eun Suk Song, David W. Rodgers, Louis B. Hersh

**Affiliations:** Department of Molecular and Cellular Biochemistry and the Center for Structural Biology, University of Kentucky, Lexington, Kentucky, United States of America; Case Western Reserve University, United States of America

## Abstract

**Background:**

Insulin degrading enzyme (IDE) is a key enzyme in the metabolism of both insulin and amyloid beta peptides. IDE is unique in that it is subject to allosteric activation which is hypothesized to occur through an oligomeric structuture.

**Methodology/Principal Findings:**

IDE is known to exist as an equilibrium mixture of monomers, dimers, and higher oligomers, with the dimer being the predominant form. Based on the crystal structure of IDE we deleted the putative dimer interface in the C-terminal region, which resulted in a monomeric variant. Monomeric IDE retained enzymatic activity, however instead of the allosteric behavior seen with wild type enzyme it displayed Michaelis-Menten kinetic behavior. With the substrate Abz-GGFLRKHGQ-EDDnp, monomeric IDE retained ∼25% of the wild type activity. In contrast with the larger peptide substrates β-endorphin and amyloid β peptide 1–40, monomeric IDE retained only 1 to 0.25% of wild type activity. Unlike wild type IDE neither bradykinin nor dynorphin B-9 activated the monomeric variant of the enzyme. Similarly, monomeric IDE was not activated by polyphosphates under conditions in which the activity of wild type enzyme was increased more than 50 fold.

**Conclusions/Significance:**

These findings serve to establish the dimer interface in IDE and demonstrate the requirement for an oligomeric form of the enzyme for its regulatory properties. The data support a mechanism where the binding of activators to oligomeric IDE induces a conformational change that cannot occur in the monomeric variant. Since a conformational change from a closed to a more open structure is likely the rate-determining step in the IDE reaction, the subunit induced conformational change likely shifts the structure of the oligomeric enzyme to a more open conformation.

## Introduction

Insulin-degrading enzyme (IDE) also known as insulysin, is a zinc metalloprotease first described based on its ability to cleave insulin [1]–[3]. Mutations in IDE are linked to a type II diabetic phenotype in the GK rat [4]. In addition IDE has been shown to play a key role in Alzheimer's disease in that it is one of the major enzymes responsible for amyloid β peptide (Aβ) clearance in the brain. Studies from this [5] and other laboratories [6] have shown that mice lacking IDE activity through gene disruption accumulate Aβ in the brain in a gene dose dependent manner. A number of studies have linked the IDE gene to both type 2 diabetes [7], [8] and AD [9], [10] although in the latter case a genetic association has yet to be firmly established.

We first reported that IDE is unique among the zinc metalloproteases in that it exhibits allosteric kinetic behavior [11]. The reaction of IDE with the internally quenched fluorogenic substrate 2-aminobenzyl-glycyl-glycyl-phenylalanyl-leucyl-arginyl-lysyl-histidyl-glycyl-asparaginyl-ethylenediamine-2,4-dinitrophenol (Abz-GGFLRKHGQ-EDDnp) was shown to exhibit both substrate induced (homotrophic) activation as well as activation produced by small peptides (heterotrophic activation). In addition we reported that polyphosphates such as ATP and triphosphate act as non-substrate heterotrophic activators, binding at a site distinct from the active site and distinct from the peptide activation site [12].

Recently the structures of IDE both liganded and unliganded were determined by Tang and co-workers [13]–[15]. These structures show that larger peptides make extended binding interactions at both the active site and at a distal site. We have recently determined the structure of rat IDE and found that this distal binding site serves as the site in which small peptide activators bind (Nicholas Noinaj, Sonia K. Bhasin, Eun Suk Song, Kirsten Scoggin, Louis B. Hersh, and David W. Rodgers, manuscript in preparation).

IDE exists as a mixture of monomers, dimers, and tetramers with the dimer the predominant species [11]. From the IDE structure of Shen et al [13] and confirmed in our IDE structure an interface between the two monomeric units involving elements from domains 3 and 4 of the four-domain protein was identified. We have taken advantage of this structural data to generate a monomeric variant of IDE. Studies on this monomeric IDE support our previous hypothesis [11] that an oligomeric form of IDE is required for allosteric activation.

## Results

**Figure 1:**
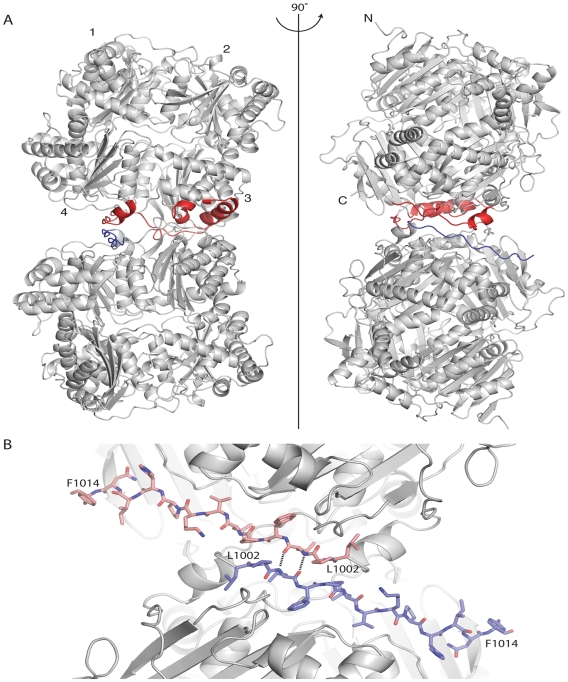
Proposed dimer of IDE. (A) Two orthogonal views of rIDE monomers from the crystal lattice are shown with elements making up the dimer interface highlighted in red for the top subunit. IDE is composed of four structurally related domains. Those domains are numbered in the top subunit in the left panel, and the N and C termini are indicated for the top subunit in the right panel. The C terminal region of IDE deleted in the rIDE^ΔC^ construct intended to destabilize the dimer interface is highlighted in blue for the lower monomers in both panels. (B) Close up of the dimer interface showing the region deleted in the rIDE^ΔC^ construct. Deleted residues 1002–1014 are shown in a stick representation with sheet-like hydrogen bonds across the interface at Leu^1004^ indicated by dashed lines.

IDE is known to exist in a monomer-dimer-tetramer equilibrium with the dimer being the predominant form [11]. We examined our crystal structure of rIDE for a possible dimerization domain and found that molecular packing within the rIDE crystal lattice consistent with the presence of a dimer interface at the C-terminal region of the enzyme. This possibility is supported by the conservation of this interface in the crystals of hIDE [13] even though the human enzyme crystallizes in a different form. The proposed dimer interface buries about 1,400 Å^2^ of accessible surface area and involves elements from domains 3 and 4 of the four structurally related domains that make up the enzyme, Figure 1. A key set of contacts occurs between regions near the C-terminus of each monomer, residues 1002–1006, which make non-polar and hydrogen bond interactions across the interface.

rIDE has only 13 residues C terminal to this interaction region, and only eight (to residue 1014) are seen in the crystal structure, which suggests that the C-terminal region of IDE could be removed without greatly compromising the stability of the enzyme. Such a deletion should destabilize the dimer interface sufficiently to make monomeric IDE the predominant form in solution. To test this hypothesis and to study the properties of monomeric IDE we generated a mutant in which residues 1002 to 1019 were deleted yielding rIDE^ΔC^. This mutant, as well as wild type rIDE, were expressed in insect cells as hexahistidine fusion proteins and purified by nickel affinity chromatography.

**Figure 2:**
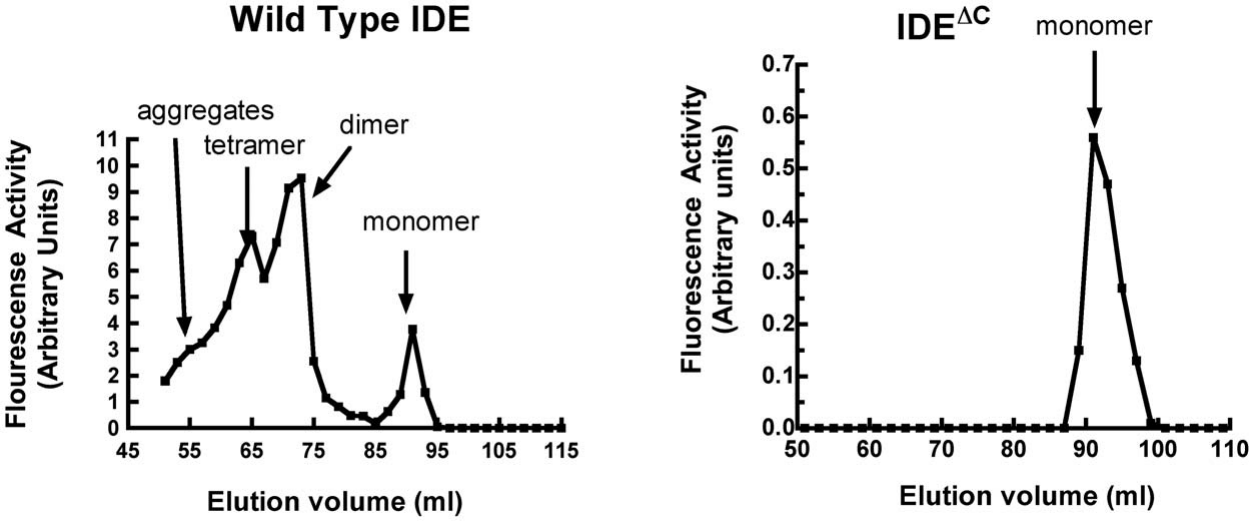
Comparison of the molecular weight forms of wild type IDE and IDE^ΔC^ on a Superdex S200 gel filtration column. Wild type IDE or the IDE^ΔC^ variant was chromatographed on a Pharmacia Superdex S200 gel filtration column in 20 mM potassium phosphate buffer, pH 7.4. One ml of protein at 0.5 mg/ml was loaded and the column developed at a flow rate of 0.4 ml/min. Fractions were collected and assayed for IDE activity using Abz-GGFLRKHGQ-EDDnp as the substrate.

We demonstrated that rIDE^ΔC^ is monomeric by molecular sieve chromatography. As shown in Figure 2, chromatography of wild type rIDE on a Superdex S200 column revealed the presence of tetrameric, dimeric, and monomeric species, with the dimer being the predominant form as expected. In contrast chromatography of rIDE^ΔC^ on the same column at the same protein concentration showed a single monomeric species.

**Figure 3:**
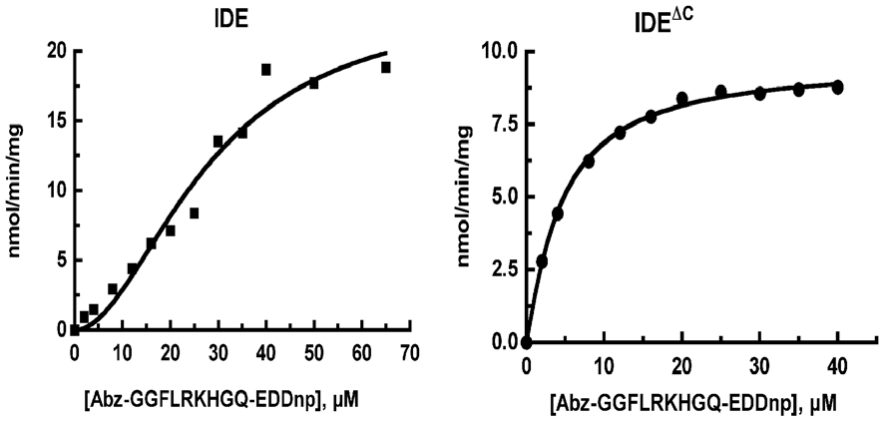
Comparison of the kinetics of IDE^ΔC^ and wild type IDE. Reactions were conducted in 50 mM Tris-HCl, pH 7.4 using either 0.25 µg of wild type IDE (left) or 1.25 µg of IDE^ΔC^ (right) and Abz-GGFLRKHGQ-EDDnp as the variable substrate. Data were fit to either a hyperbolic or sigmoidal response curve as described in Methods with the values and errors included in Table 1.

The kinetics of the rIDE^ΔC^ reaction was compared to wild type rIDE using the fluorogenic substrate Abz-GGFLRKHGQ-EDDnp. rIDE^ΔC^ retained enzymatic activity, however it no longer exhibited the sigmoidal kinetics seen with wild type rIDE, Figure 3. The kinetic constants, summarized in Table 1, show that rIDE^ΔC^ exhibits about 25% of the k_cat_ of wild type rIDE, but has a lower substrate K_M_. In contrast to wild type rIDE, where the reaction is cooperative with a Hill coefficient of 2, rIDE^ΔC^ exhibits classical Michaelis-Menten kinetics with a Hill coefficient of ∼1.

**Table 1:** Comparison of kinetic constants for IDE and monomeric IDE.

	IDE	IDE^ΔC^
k_cat_ (min^−1^)	2.62±0.26	0.67±0.02
K_0.5_ or K_m_ (µM)	21.5±3.7	6.6±0.2
Hill coefficient	2.0±0.3	1.1±0.1

Kinetic constants were derived by fitting the data from two experiments similar to that shown in figure 3 to a sigmoidal or hyperbolic substrate versus velocity response curve as described in Methods.

**Figure 4:**
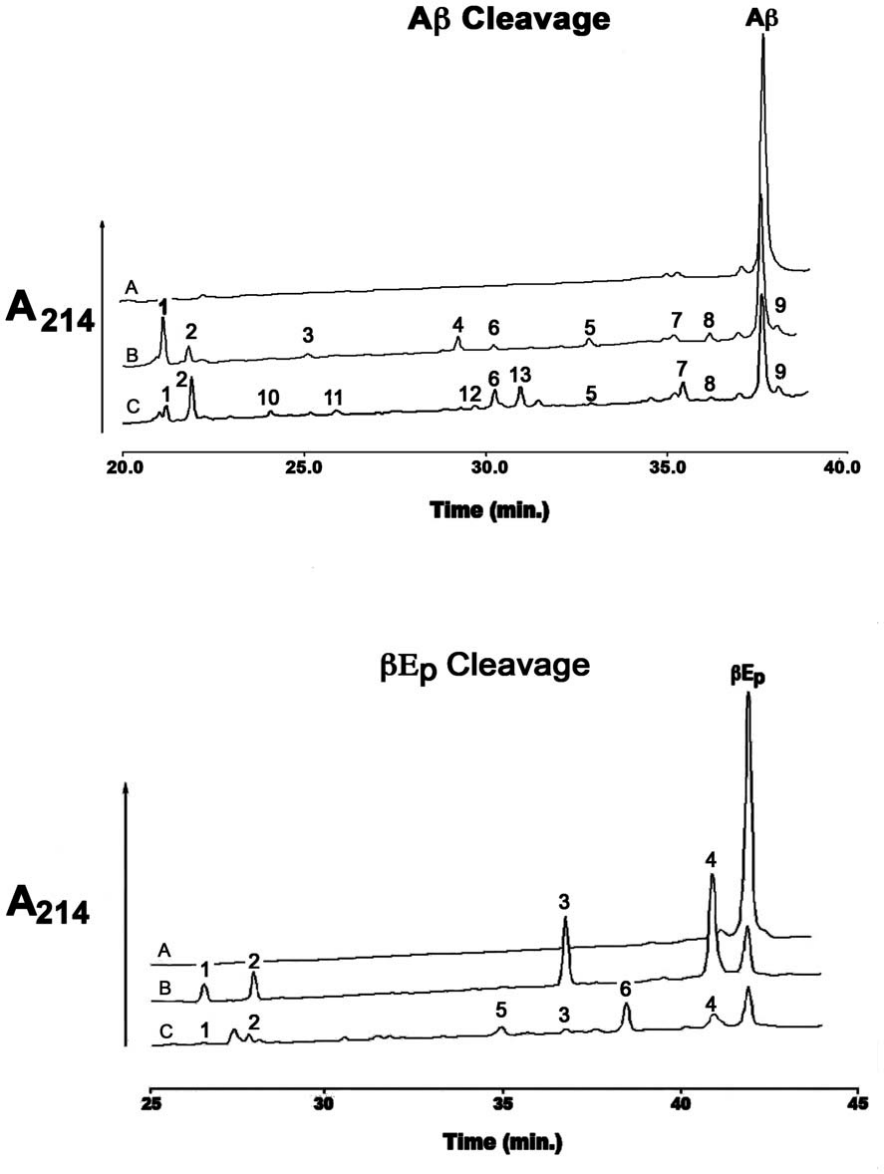
HPLC chromatograms showing the cleavage of Aβ_1–40_ and β-endorphin by IDE and IDE^ΔC^. (Top) Reaction mixtures containing 10 µM Aβ_1–40_ in 50 mM Tris-HCl, pH 7.4 (curve A) were incubated with 100 ng of IDE for 5 min (curve B) or 2 µg of the monomeric IDE variant for 3 hrs (curve C). The amount of enzyme and time used was chosen so that a similar fraction of substrate would be consumed by both IDE and the monomeric IDE variant permitting a comparison of products formed at the same fraction of substrate consumed. Reaction products were separated by gradient HPLC on a Vydac C4 reverse phase column as previously described (16). Product peaks were collected manually and identified by mass spectrometry. Peak 1 is Aβ_1–14_ (observed mass = 1698.72, calculated mass = 1698.72); peak 2 is Aβ_1–13_ (observed mass = 1561.68, calculated mass = 1561.58); peak 3 is Aβ_1–18_ (observed mass = 2167.32, calculated mass = 2167.32); peak 4 is Aβ_1–13_ (observed mass = 2314.50, calculated mass = 2314.5); peak 5 is Aβ_1–20_ (observed mass = 2461.67, calculated mass = 2461.67); peak 6 is Aβ_5–28_ (observed mass = 1581.78, calculated mass = 1581.78); peak 7 is Aβ_21–40_ (observed mass = 1885.96, calculated mass = 1886.2); peak 8 is Aβ_20–40_ (observed mass = 2033.02, calculated mass = 2033.37); peak 9 is Aβ_4–40_ (observed mass = 2785.50, calculated mass = 2786.29); peak 10 is Aβ_1–17_ (observed mass = 2068.04, calculated mass = 2068.19); peak 11 is Aβ_26–40_ (observed mass = 1413.80, calculated mass = 1414.73); peak 12 is Aβ_19–33_ (observed mass = 1524.76, calculated mass = 1524.69); peak 13 is Aβ_14–22_ (observed mass = 1118.60, calculated mass = 1118.3). Note peaks 3 and 4 are absent in the rIDE^ΔC^ cleavage products, while peaks 10 to 13 are unique to rIDE^ΔC^. Although not shown the reaction was followed from ∼15% cleavage to ∼90% cleavage with similar results. (Bottom) Reaction mixtures containing 10 µM β-endorphin (βEp) in 50 mM Tris-HCl, pH 7.4 (curve A) were incubated with 50 ng of IDE for 15 min (curve B) or 2 µg of the monomeric IDE variant for 3 hrs (curve C). Reaction conditions and product separation and identification were as in (A) above. Peak 1 is β-endorphin 19–31 (observed mass = 1475.84, calculated mass = 1476.74); peak 2 is β-endorphin 18–31 (observed mass = 1623.91, calculated mass = 1623.91); peak 3 is β-endorphin 1–17 with methionine oxidized to its sulfoxide (observed mass = 1874.94, calculated mass = 1875.13); Peak 4 is β-endorphin 1–18 (observed mass = 2005.97, calculated mass = 2006.3); peak 5 is β-endorphin 7–20 (observed mass = 1590.86, calculated mass = 1591.82); peak 6 is β-endorphin 7–18 (observed mass = 1348.70, calculated mass = 1349.54). Note peaks 5 and 6 were only observed in the reaction with IDE^ΔC^. Although not shown the reaction was followed from ∼20% cleavage to ∼85% cleavage with similar results.

We next determined whether rIDE^ΔC^ retained the ability to degrade larger physiological substrates by comparing its reaction with β-endorphin and amyloid β peptide 1–40 (Aβ_1–40_) to the reaction of wild type rIDE. At a fixed concentration of 10 µM substrate rIDE^ΔC^ cleaved these larger peptide substrates at about 1 to 0.25% the rate of wild type rIDE. With Aβ_1–40_ we observed peptide products that appeared with wild type IDE, but were absent from the rIDE^ΔC^ cleavage, and unique products that appeared only in the rIDE^ΔC^ cleavage. As shown in figure 4 (top), those products absent in the rIDE^ΔC^ cleavage of Aβ_1–40_ were identified as Aβ_1–18_ (cleavage at a Val-Phe bond) and Aβ_1–13_ (cleavage at a His-His bond), while products unique to the rIDE^ΔC^ cleavage of Aβ_1–40_ were Aβ_1–17_ (unique cleavage at a Leu-Val bond), Aβ_14–22_ (unique cleavage at a Glu-Asp bond), Aβ_19–33_ (unique cleavage at a Gly-Leu bond), and Aβ_26–40_ (unique cleavage at Ser-Asn bond).

Similarly we identified two additional cleavage sites in β-endorphin with rIDE^ΔC^, Figure 4B. IDE normally cleaves β-endorphin at the Leu^17^-Phe^18^ and Phe^18^-Lys^19^ bonds [16]. The rIDE^ΔC^ mutant cleaved at these sites, but in addition cleaved at Thr^6^-Ser^7^ plus Phe^18^-Lys^19^ and at Thr^6^-Ser^7^ plus Asn^20^-Ala^21^. Time course experiments showed that these products could not be detected with wild type IDE throughout the entire time course of its reaction with β-endorphin. Additional cleavage sites in β-endorphin (Met^5^-Thr^6^ and Lys^19^-Asn^20)^ were previously observed in the reaction of rIDE mutants containing substitutions of the active site glutamate [16].

**Figure 5:**
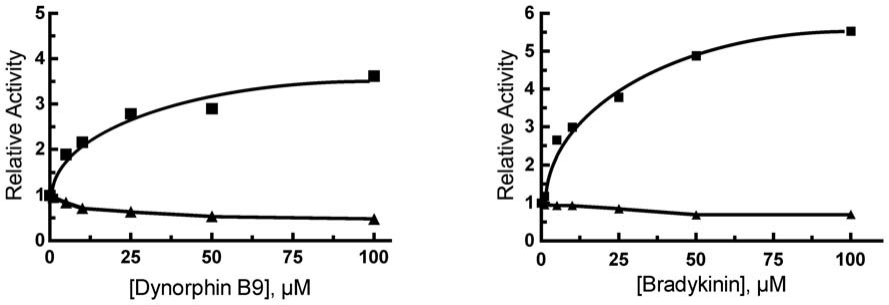
Effect of bradykinin and dynorphin B9 on the reaction of IDE^ΔC^ and wild type IDE with Abz-GGFLRKHGQ-EDDnp. Activity was determined in 50 mM Tris-HCl, pH 7.4 with 10 µM Abz-GGFLRKHGQ-EDDnp as substrate and the indicated concentrations of bradykinin (left) or dynorphin B9 (right). The reactions with wild type IDE (filled squares) contained 0.25 µg of protein and with IDE^ΔC^ (filled triangles) 1.25 µg of protein was used. With wild type IDE the maximal activation produced by bradykinin varied from 4.4 to 5.4 fold, while a 15 to 30% inhibition was seen with monomeric IDE. Similarly dynorphin B9 produced a 3.4 to 4.3 fold activation of wild type IDE, but inhibited the monomeric IDE variant 10 to 40%.

We previously reported that substrates for IDE such as bradykinin and dynorphin B-9 act as activators of the hydrolysis of Abz-GGFLRKHGQ-EDDnp [11]. Based on our hypothesis that activation of IDE by activators occurs through conformational changes transmitted from one subunit to the other, we would predict that the monomeric rIDE^ΔC^ variant would not exhibit this activation. That this is the case is shown for bradykinin and dynorphin B9 as the effectors, Figure 5. With wild type rIDE, bradykinin and dynorphin B-9 activate the reaction while with rIDE^ΔC^ these peptides inhibited the reaction slightly.

**Figure 6:**
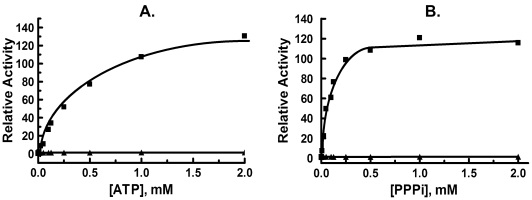
Effect of ATP and PPPi on the reaction of IDE^ΔC^ and wild type IDE with Abz-GGFLRKHGQ-EDDnp. Reactions contained 50 mM Tris-HCl, pH 7.4 with 10 µM Abz-GGFLRKHGQ-EDDnp as substrate and the indicated concentrations of ATP (A) or PPPi (B). The reactions with wild type IDE (filled squares) contained 0.25 µg of protein while the reaction with IDE^ΔC^ (filled triangles) contained 1.25 µg of protein. In separate experiments ATP increased the activity of wild type IDE 130 to 160 fold, while PPPi increased the rate 88 to 97 fold. In contrast ATP increased the rate of the monomeric IDE variant 1.5 to 3 fold, while PPPi increased the rate 1.5 to 2.5 fold.

**Figure 7:**
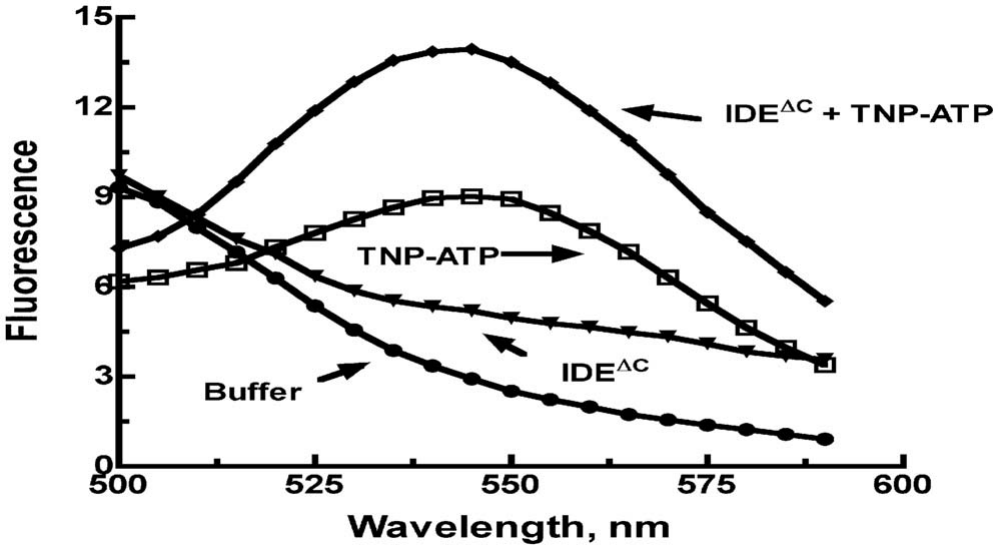
Binding of TNP-ATP to IDE^ΔC^. Fluorescence emission spectra of 10 µM TNP-ATP in the presence or absence of 1.5 µM of IDE^ΔC^ in 50 mM Tris-HCl, pH 7.4 were recorded on a Perkin-Elmer LS 55 Luminescence spectrometer. Fluorescence spectra were recorded with a λ_exc_ = 403 nm.

Polyphosphates, including ATP and triphosphate (PPPi) also activate the IDE dependent hydrolysis of small substrates including Abz-GGFLRKHGQ-EDDnp [12]. The ATP binding site was shown to be distinct from both the substrate binding site as well as the distal site where peptide activators such as bradykinin and dynorphin B-9 bind. We thus determined whether ATP or PPPi [12] were capable of increasing the rate of the reaction of rIDE^ΔC^ with Abz-GGFLRKHGQ-EDDnp. As shown in Figure 6 neither ATP nor PPPi could activate the reaction of rIDE^ΔC^ under conditions in which the reaction of the wild type enzyme was increased more than 100 fold. Interaction of the ATP analog TNP-ATP with IDE can be followed by measuring the change in fluorescence that occurs upon binding [17]. We therefore compared the fluorescence spectra of TNP-ATP in the presence and absence of the monomeric IDE variant. As shown in Figure 7, the monomeric IDE variant produces an increase in TNP-ATP fluorescence and a slight shift in the fluorescence maximum to a lower wavelength, characteristic of TNP binding [17]. However the shift in fluorescence maximum is notably less than observed with wild-type IDE.

## Discussion

Contacts in crystals of IDE suggest that the dimer interface is formed by elements of the two C-terminal domains, including a sheet-like contact formed by a terminal β strand. Thus by deleting 18 residues from the C terminus, we anticipated that the intermolecular contacts would be sufficiently weakened that only monomers would be stable. This was indeed the case as evidenced by the finding of only monomeric rIDE in the deletion mutant preparation.

The monomeric rIDE variant exhibits 25% of the catalytic activity of wild type rIDE and no longer exhibits substrate induced homotrophic activation with Abz-GGFLRKHGQ-EDDnp. Substrate induced cooperativity or homotrophic activation, likely results from binding of the substrate at a site distinct from the catalytic site that is part of an extended substrate-binding site (Nicholas Noinaj, Sonia K. Bhasin, Eun Suk Song, Kirsten Scoggin, Louis B. Hersh, and David W. Rodgers, manuscript in preparation). Binding at this region of the extended substrate binding site is suggested to produce a conformational change in the adjacent subunit. Thus the absence of substrate induced cooperativity as shown by change from a sigmoidal substrate versus velocity response seen with wild type IDE to a hyperbolic response seen in monomeric IDE can be explained by the loss of subunit-subunit interactions.

In contrast to the relatively small decrease in k_cat_ observed with Abz-GGFLRKHGQ-EDDnp as substrate, the monomeric rIDE variant exhibits a 100 to 200-fold decrease in the rate of cleavage of the larger physiological peptides β-endorphin and amyloid β peptide. Larger peptides like β-endorphin and amyloid β peptide traverse the protein and bind both at the active site as well as at a distal site [13]. Since a portion of amyloid β peptide (and likely β-endorphin) occupies the distal site we suggest these substrates “self-activate” producing the same or similar conformational change in the adjacent subunit and are thus insensitive to heterotrophic activation.

The crystallographic studies of Tang and coworkers [13] show that IDE totally engulfs the bound peptide in the Michaelis complex, with no room for products to dissociate. Thus the rate-determining step must involve a conformational change that opens the enzyme to permit product dissociation. The increase in k_cat_ produced by activators with small synthetic substrates would therefore involve an increase in the rate of enzyme opening produced by the binding of substrate to one subunit and transmitting a conformational change to the adjacent subunit. Clearly this cannot occur in monomeric IDE, leading to a decrease in kcat.

With the larger substrates β-endorphin and amyloid β peptide the extended binding interactions would be expected to make the conformational change needed for product release energetically more difficult than with small substrates. We suggest that one possibility is that hydrolysis of the substrate in the active site of one oligomeric subunit produces the driving force that leads to a conformational change transmitted to the adjacent subunit. This conformational change increases the rate of enzyme opening and product release. In the monomeric enzyme the absence of an induced conformational change would be a major contributing factor to the slow rate of cleavage of physiological peptide substrates. An alternative hypothesis is that substrate binding in itself is sufficient to translate a conformation change from one monomeric unit to the other which shifts the equilibrium of one subunit to the open conformation and promotes product release.

The observation that both Aβ_1–40_ and β-endorphin are cleaved at unique sites by monomeric rIDE further suggests that substrate binding is different in the monomeric enzyme. Thus interactions between subunits in the oligomeric form of IDE likely contribute to the conformation of the enzyme-substrate complex.

It should be noted that we can not rule out a mechanism in which dimer contacts are required for activation to occur within a subunit, although previous studies [11] with mixed dimers containing one catalytically active and one catalytically inactive subunit weigh against this alternative. It is also possible that loss of the C-terminal 18 residues directly affects the properties of the enzyme independent of the effect on oligomerization. Given the tenuous attachment of these residues to the rest of the enzyme, this possibility seems unlikely.

We find that in contrast to wild type IDE, the activity of monomeric IDE is not increased by small peptides or by polyphosphates. However binding of the nucleotide triphosphate TNP-ATP does occur as shown by an increase in its fluorescence in the presence of monomeric IDE. Thus the absence of activation of monomeric IDE in the presence of binding can be accounted for by the necessity of a conformational change induced by activator binding to one subunit and transmitted to the adjacent subunit. Alternatively or additionally the oligomeric form of IDE might be required for proper activator binding. That this may be a contributing factor is indicated by the observation of a smaller shift of the fluorescence maxima of bound TNP-ATP with monomeric IDE compared to oligomeric IDE.

## Materials and Methods

### Preparation of an IDE C-terminal Deletion Mutant

For construction of an IDE mutant with the C-terminal 18 amino acids deleted PCR was used to generate a 360 bp fragment containing a stop codon, which replaced a 415 bp Pst I – Xho I fragment from the rIDE cDNA in pFast Bac HTb.

Oligonucleotides used for preparing the deletion mutant were:

5′ – TATCTGCAGAGTGCGCGAAGTACTAGG – 3′

Pst I

5′ – AGACTCGAG**TCA**GCCGCGCTTGAATTCAGT – 3′

Xho I stop codon

### IDE Expression and Purification

Native rIDE and its C-terminal deletion mutant, rIDE^ΔC^ were expressed in Sf9 insect cells and purified as hexahistidine fusion proteins on HIS-select Ni-NTA agarose (Sigma) as previously described [16], [18]. This procedure generally yielded homogeneous native rIDE, however in those instances when contaminants were detected, anion exchange chromatography on a 1 ml MonoQ column was used to produces enzyme of ∼90% purity. Recombinant rIDE^ΔC^ did not express as well as wild type rIDE in Sf9 cells and yielded enzyme preparations of ∼50–60% purity.

### Enzyme Activity Assay

The internally quenched fluorogenic peptide Abz-GGFLRKHGQ-EDDnp was used to routinely measure IDE activity as previously described [18]. Reaction mixtures (200 µl) contained 50 mM Tris-HCl, pH 7.4 with 10 µM Abz-GGFLRKHGQ-EDDnp.

### Kinetic Analysis

Kinetic data were fit using Graphpad software to either a hyperbolic substrate versus velocity response curve (v = V_max_[S]/(K_M_ + [S]) (Michaelis-Menten equation) or to a sigmoidal response curve (v = V_max_[S]^h^/(K_M_ + [S]^h^), where v is the observed rate, V_max_ is the maximal velocity, K_M_ is the Michaelis constant, S is the variable substrate, and h is the Hill coefficient. For reactions with physiological peptides, a fixed concentration of β-endorphin (10 µM) and amyloid β peptide (10 µM) was incubated with IDE (50 ng) or IDE^ΔC^ (2 µg) for 15 min to 3 hrs. The rate of peptide cleavage was determined by following the disappearance of the parent peptide by HPLC [16]. Product peaks were collected and their identification determined by tandem mass spectrometry.

### Activation studies

Activation of IDE by peptides or by triphosphates was determined by measuring their ability to increase the rate of Abz-GGFLRKHGQ-EDDnp cleavage. Data were fit to a hyperbolic response curve using Prism Graphpad software.

### Molecular weight determination

Gel filtration was performed on a Pharmacia Superdex S200 column equilibrated with 20 mM potassium phosphate buffer, pH 7.4 and run at a flow rate of 0.4 ml/min. The eluted fractions (1 ml) were assayed for IDE activity using Abz-GGFLRKHGQ-EDDnp as substrate.
